# Genomic identification of *Oryctes rhinoceros* nudivirus isolates, a biocontrol agent for coconut rhinoceros beetle

**DOI:** 10.1007/s00203-024-04116-y

**Published:** 2024-09-26

**Authors:** Ela Hiszczynska-Sawicka, Mitchell K. Weston, Aurelie Laugraud, Charles A. Hefer, Jeanne M. E. Jacobs, Sean D. G. Marshall

**Affiliations:** AgResearch Ltd., 19 Ellesmere Junction Road, Lincoln, 7674 New Zealand

**Keywords:** CRB, Diagnostics, Isolates, Nanopore sequencing, Oryctes nudivirus, *Oryctes rhinoceros*

## Abstract

The coconut rhinoceros beetle (*Oryctes rhinoceros*, CRB) is a serious pest of coconut and oil palms. It is native to South and Southeast Asia and was inadvertently introduced to Samoa in 1909. It has invaded many other Pacific countries throughout the last century. *Oryctes rhinoceros* nudivirus (OrNV), a natural pathogen of CRB in its native range, was successfully introduced as a classical biocontrol agent and has effectively suppressed invasive CRB populations for decades. However, resurgence of CRB has been recorded, with new invasions detected in several Pacific Island Countries and Territories. Additionally, new populations of CRB are emerging in some invaded areas that have a degree of resistance to the virus isolates commonly released for CRB biocontrol. Here, we designed a fast and reliable tool for distinguishing between different OrNV isolates that can help with the selection process to identify effective isolates for management of new CRB invasions. A comparison of 13 gene/gene region sequences within the OrNV genome of 16 OrNV isolates from native and invaded ranges allowed us to identify unique Single Nucleotide Polymorphisms (SNPs). With these SNPs, we developed an assay using multiplex PCR-amplicon-based nanopore sequencing to distinguish between OrNV isolates. We found that as few as four gene fragments were sufficient to identify 15 out of 20 OrNV isolates. This method can be used as a tool to monitor the establishment and distribution of OrNV isolates selected for release as biocontrol agents in CRB-infected areas.

**Supplementary Information:**

The online version contains supplementary material available at 10.1007/s00203-024-04116-y.

## Introduction

*Oryctes rhinoceros* (L.) (Coleoptera: Scarabaeidae), known as the coconut rhinoceros beetle (CRB), is a serious pest of coconut and oil palms in tropical Asia and the Pacific. Adult CRB bore into the crown of coconut palms to access the sap as a food source. Severe, repeated attacks kill the apical meristem, resulting in the death of the coconut palm (Bedford 2013). In oil palm, adult CRB primarily attack young palms, boring into the base of the developing fronds, and repeated attacks can kill young palms and severely reduces yields in older palms (Manjeri et al. 2014). Furthermore, secondary infestation by other insects (e.g., *Rhynchophorus* spp.) via CRB bore holes can also contribute to palm mortality (Bedford 2013). In contrast, CRB larvae feed on decaying organic matter and do not cause palm damage.

During the twentieth century, human-mediated dispersal resulted in the distribution of CRB expanding from its native range between Pakistan and the Philippines (Catley 1969). Outside this region, CRB was accidentally introduced to a few islands of the Pacific and Indian Oceans and then spread rapidly within several Pacific Island Countries and Territories (PICTs) (reviewed in Paudel et al. 2021). The first report of CRB in the Pacific region was from Upolu, Samoa in 1909 (Catley 1969). The *Oryctes rhinoceros* nudivirus [*Alphanudivirus oryrhinocerotis* (van Oers et al. 2023); abbreviated as OrNV], a virus with a circular dsDNA genome, was first isolated in 1963 from Malaysia, within CRB’s native range (Huger 1966; Gopal 2001). This original Malaysian OrNV (collected from an unspecified locality) became a successful classical biocontrol agent against CRB in the South Pacific Islands (Bedford 1980). OrNV is infectious to both larval and adult stages of CRB, although transmission is most effective between adults under natural conditions (Bedford 2013).

The OrNV isolate from Malaysia was first released as a biocontrol agent against CRB in Samoa in 1967 (Marshall 1970). It became established within one year and spread to other parts of Samoa. Subsequent releases of OrNV were made on Wallis Island, Tonga, Fiji, Tokelau, Palau, American Samoa, Papua New Guinea (Catley 1969; Bedford 1976; Schreiner 1989; Hajek et al. 2021; Gorick 1980) as well as the Maldives (Zelazny et al. 1990, 1992) and India (Jackson 2009). In the PICTs where OrNV was released it was found to have established within approximately 1 to 3 years and spread through the invasive CRB populations. This resulted in a significant decline in palm damage, e.g. from 80 to 36% in Caboni, Fiji (summarized in Bedford 1980). For over 35 years the introduction of OrNV into the Pacific Islands was a major success in reducing extreme outbreak populations of CRB and resolving the critical problem of managing beetle damage to palms. However, in the last two decades, there has been a resurgence of CRB*,* with invasions being recorded in new PICTs (Jackson et al. 2005; Jackson 2009; Marshall et al. 2017).

Previous studies have indicated that the efficiency of damage reduction is influenced by the particular OrNV isolate released as a biocontrol agent (Zelazny 1979; Zelazny et al. 1990). For example, among three isolates (X2B, S2A and V23B) introduced to the Mulaku group of islands (Maldives) in 1984, isolate X2B was found to be the most prevalent four years later (Crawford and Zelazny 1990; Zelazny et al. 1990). This was not a surprise, as isolate X2B was previously characterized as a highly active isolate (Crawford and Zelazny 1990). Nevertheless, a balance between virulence and transmission is an important consideration; a level of virulence that is too high may result in the eradication of only the local population, while low virulence is unlikely to provide enough control. More recent work has suggested that CRB susceptibility to OrNV varies depending on the combination of the specific OrNV isolate and host CRB population (e.g. Marshall et al. 2017; Reil et al. 2018; Tanaka et al. 2021). Therefore, the sensitivity of the beetle population to various OrNV isolates must be assessed to choose the best isolate that can balance virulence and infectivity (Zelazny 1979). Screening for more effective virus isolates to manage these newer invasions is essential for classical biological control of CRB.

Since the discovery of OrNV, detection of infection status has been based on the visual observation of the dissected beetle midgut (i.e. presence or absence of a white and swollen midgut), histology of the midgut and/or electron microscopy (Marschall and Ioane 1982; Zelazny et al. 1992). Genetic variation in OrNV field isolates collected from different geographic locations was initially detected using a restriction fragment length polymorphisms (RFLP) assay with purified viral DNA (Crawford et al. 1986). Based on subtle changes in virus DNA fragment lengths of the respective RFLP profiles, the authors distinguished between the 12 OrNV isolates that were analysed; however, this method is time-consuming and requires a significant amount of purified virus DNA (Crawford et al. 1986). A PCR-based method developed subsequently (Richards et al. 1999) can detect presence of OrNV before infection symptoms are noticeable but is unable to identify specific OrNV isolates associated with infection. Therefore, development of faster and more precise methods to distinguish between OrNV isolates is needed.

DNA sequencing has played a critical role in improving understanding of the genetic diversity, molecular epidemiology, and transmission chain of several virus outbreaks (Lee et al. 2022; Grubaugh et al. 2019). While DNA Sanger sequencing was used to generate the first complete OrNV genome sequence (Wang et al. 2008), further advancements in DNA sequencing technology are now allowing for the rapid generation of whole genome sequences of multiple isolates of OrNV. As a result, detailed analyses of genetic variation at the nucleotide level, including patterns of variation, are starting to emerge (Tanaka et al. 2021; Etebari et al. 2020; Weston et al. 2023).

More modern DNA sequencing technologies can enable much faster identification, require less material and increase the throughput of samples (Shaw et al. 2023; Chen et al. 2023). For example, the Oxford Nanopore Technologies (ONT) nanopore sequencing platform offers rapid and low-cost DNA sequencing technology. A portable device for real-time sequencing (e.g. MinION, ONT, UK) is highly suited for use in field situations or hospitals (e.g. Zika virus, SARS-CoV-2) (Faria et al. 2016; Lawley et al. 2021). The barcoding of PCR amplicons enables simultaneous sequencing of multiple samples, reducing the per-sample cost and time required to obtain results. A multiplex, conventional PCR assay has frequently been used to distinguish between early life stages of insects or even their faecal excrement as well as to identify different insect species (e.g. thrips) (Watanabe and Melzer 2017; Yeh et al. 2014). This method is usually combined with standard electrophoresis size separation rather than DNA sequencing.

We combined multiplex PCR with nanopore sequencing which enables the sequencing of multiple templates from multiple individuals. Multiplex PCR-based nanopore sequencing has been used for genomic surveillance of pathogens and development of control programs (Holzschuh et al. 2024) and to obtain nearly complete genome sequences of some viruses (e.g. SARS-CoV-2, Brinkmann et al. 2021).

Here, we describe the development of a new method that enables the differentiation of OrNV isolates based on unique Single Nucleotide Polymorphisms (SNPs) using multiplex PCR amplicon-based nanopore sequencing. The method can also detect new isolates appearing in the field, novel mutations or even mixed infections. Such a rapid and accurate diagnostic method for virus identification would facilitate the monitoring process for OrNV in the field and help guide CRB management decisions. This can include monitoring virus spread following a biocontrol release.

## Materials and methods

### Viral isolates

OrNV was isolated from virus-infected CRB samples collected from different geographic locations in Asia and the Pacific Islands between 1977 and 2015 (Crawford et al. 1986; Crawford and Zelazny 1990; Zelazny et al. 1990; Crawford 1982; Marshall et al. 2017) (Table 1). Viral samples were multiplied in the African black beetle (*Heteronychus arator*) cell line DSIR-HA-1179 (Crawford 1982) according to the procedure described by Crawford and Sheehan (1985). Individual purified virus isolates were stored at 4 °C in the AgResearch Microbial Culture Collection and when required, the viral stocks were refreshed by a passage in the *Heteronychus arator* DSIR-HA-1179 cell line as described in detail in Marshall et al. (2017) and Pushparajan et al. (2017).

**Table 1:** Synopsis of *Oryctes rhinoceros* nudivirus (OrNV) isolates included from the AgResearch Microbial Culture Collection

	Isolate	Location	Year acquired	GenBank Acc. No	References
1	22A	Southern Luzon, Philippines	1983		Crawford et al. (1986)
2	46P	Southern Luzon, Philippines	1983		Crawford et al. (1986)
3	AP371	Southern Luzon, Philippines	1983		Crawford et al. (1986)
4	B36	Bugsuk Island, Palawan, Philippines	1984		Crawford et al. (1986)
5	Dug42	Dumaguete, Philippines	2016	OP831186	Weston et al. (2023)
6	FJ01	Suva, Fiji	2015		
7	I	Kerala, India	1983		Crawford et al. (1986)
8	MSA	Malaysia A	1984		Crawford et al. (1986)
9	NSB	Northern Sulawesi, Indonesia	1988		
10	PNG	Rabaul, Papua New Guinea	1988	OP831187	Weston et al. (2023)
11	S2A	Tanzania	1981	OP831189	Crawford et al. (1986), Weston et al. (2023)
12	SEY	Seychelles	Unknown		Crawford et al. (1986)
13	TAP/TAS	Upolu, Samoa	2010		Marshall et al. (2017)
14	V23B	Southern Luzon, Philippines	1980	OP831190	Crawford et al. (1986), Weston et al. (2023)
15	X2B	Bugsuk Island, Palawan, Philippines	1983	MW298153	Crawford et al. (1986), Tanaka et al. (2021)
16	XMS	Unknown	Unknown		
17	YFC3	Unknown	Unknown		

### Viral DNA extraction

The Isolate II Genomic DNA Kit (Bioline, Meridian Biosciences, UK) was used to purify OrNV DNA following the manufacturer’s instructions. Briefly, for each virus isolate, a 400 µL aliquot of purified viral suspension isolated from cell culture was mixed with 400 µL of buffer GL and 100 µg of Proteinase K, vortexed for 20 s, and then incubated at 70 °C for 15 min. Following incubation, 420 µL of 98% ethanol was added to the lysates, which were then loaded onto the Isolate II Genomic DNA Spin Column (Bioline, Meridian Biosciences, UK) and processed according to the manufacturer’s protocol. DNA was eluted in 100 µL Elution Buffer G (70 °C) and stored at −20 °C.

### Total DNA extraction from field samples of CRB

Total DNA was extracted from CRB midgut tissue (where virus is most prevalent) preserved in mono propylene glycol according to Marshall et al. (2017). DNA was isolated using the Geneaid Tissue Genomic DNA Mini Kit (Geneaid Biotech, Taiwan) column system following the manufacturer’s instructions. DNA elution was carried out using 100 μL of elution buffer, with aliquots of eluted DNA samples stored at −20 °C for further analyses.

### Single nucleotide polymorphism identification and PCR primer design for OrNV diagnostics

**Fig. 1:**
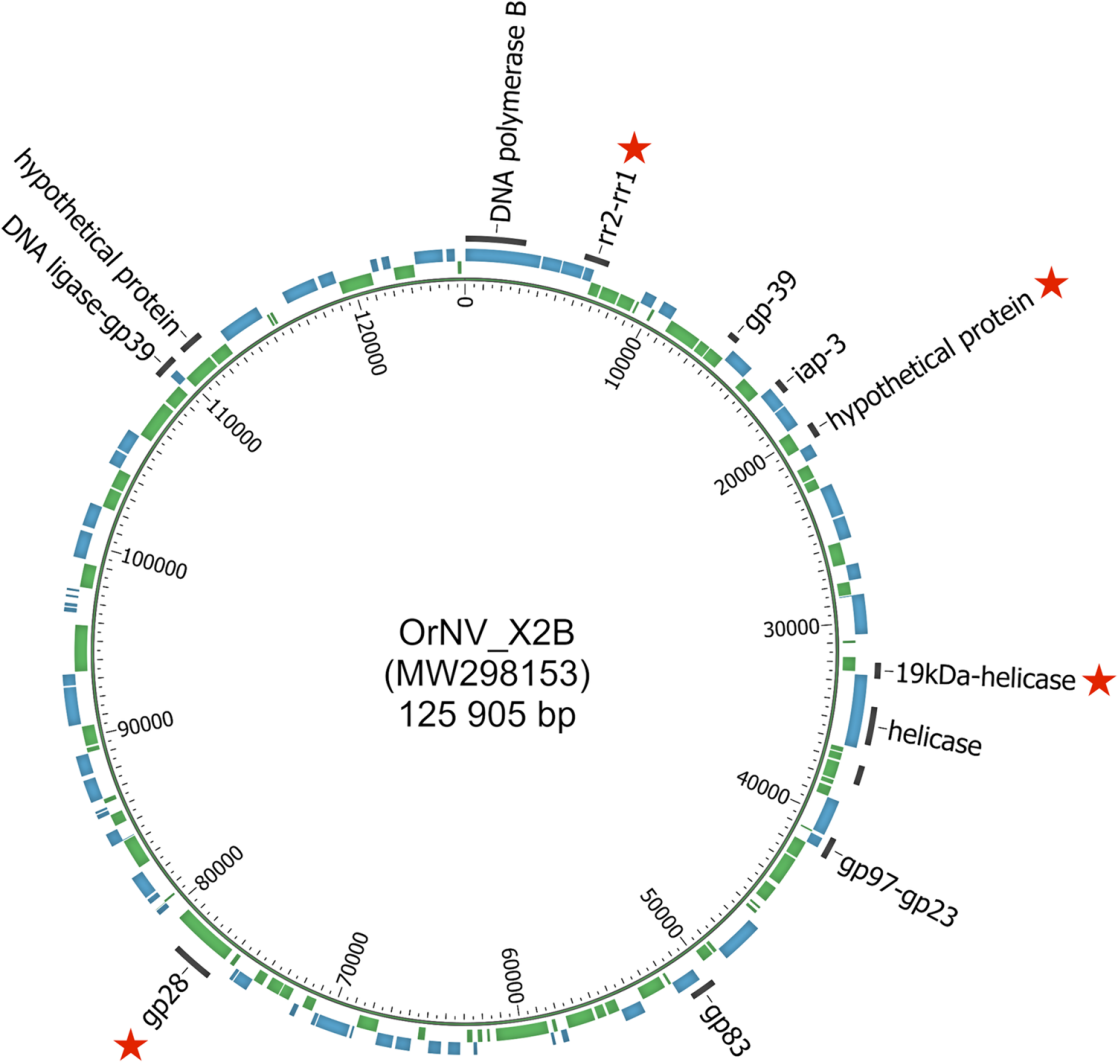
Thirteen OrNV gene regions targeted for the development of DNA markers to differentiate between OrNV isolates are displayed (with the gene names listed). *Green* and *blue bars* represent different genes. Refer to Table 2 for genomic coordinate locations. The genome of OrNV isolate X2B (MW298153) was visualized in Circos (Krzywinski et al. 2009). Selected genes/regions for OrNV isolate identification with nanopore sequencing are marked (*red stars*)

Published OrNV genome sequences were aligned using Geneious Prime® 2023.0.4 software (Kearse et al. 2012). These are NC_011588 (Ma07, Malaysia); AH015832 (PV505, Philippines); MZ727584 (LiboV, Indonesia); MN623374 (genome sequence derived from total DNA infected CRB midgut tissue, Solomon Islands); MW298154 (Palau1, Palau) and MW298153 (X2B, Philippines). Additionally, complete genome sequences of two isolates from the AgResearch Microbial Culture Collection were also included in the analysis: V23B (OP831190) and S2A (OP831189) (Weston et al. 2023) (Table 1). To find unique polymorphisms that would allow different isolates to be distinguished, we assessed the level of variation within individual genes/regions based on the alignment of the eight available OrNV genome sequences. The most highly variable regions (ranging from 585 to 1322 bp) containing unique SNPs within each gene/location were identified by visual inspection as potential sites to develop identification markers for specific OrNV isolates. A total of 19 different regions, representing 13 genes (Fig. 1), were selected to design OrNV identification primers (Table 2). Primers 18–26 nucleotides long were designed manually or using Primer 3 2.3.7 within Geneious Prime, taking care to avoid hairpins, self-dimers, and primer-dimers. Genes longer than 1500 bp were amplified in either two (helicase, gp28) or five (DNA polymerase B gene) overlapping fragments (Table 2).

**Table 2:** Primers used for OrNV selected genes amplification

Gene	Gene region	Primer name	Primer sequence 5ʹ–3ʹ	Location^a^	Amplicon size
1	gp-39^b^	OrNV_p39_F^b^	TTCATTATTCGCGTGCAGCC	14,446–14,465	585
		OrNV_p39_R^b^	AATCGGAAGAGTCGTTGCGT	13,881–13,900	
2^c^	DNA polymerase B (fragment 1)	OrNV_dnapolB_f1_F	TATCGTGGTAATAAAGAGTATTTTG	3–27	856
		OrNV_dnapolB_f1_R	CACTTTTATCGGGTATAACATCG	836–858	
	DNA polymerase B (fragment 2)	OrNV_dnapolB_f2_F	GATGATGGATACGATGTTATACCCG	825–849	848
		OrNV_dnapolB_f2_R	GACTTTTTAGACGAATATCGAGCG	1649–1672	
	DNA polymerase B (fragment 3)	OrNV_dnapolB_f3_F	CGTCTAAAAAGTCGCAATTTCCAG	1660–1683	853
		OrNV_dnapolB_f3_R	TCAACGCCAAACACATCAGC	2493–2512	
	DNA polymerase B (fragment 4)	OrNV_dnapolB_f4_F	CGCTGATGTGTTTGGCGTTG	2492–2511	727
		OrNV_dnapolB_f4_R	GCACTCGGCCCATTTACATTC	3197–3217	
	DNA polymerase B (fragment 5)	OrNV_dnapolB_f5_F	CGCTTCGGCACAATATTGTCG	2984–3004	843
		OrNV_dnapolB_f5_R	CGGTGTGAATTGGTTCGGTG	3797–3816	
3	rr2-rr1	OrNV_rr2_rr1_F	TGGGCGGTACATCATCATCG^d^	5871–5890	1282
		OrNV_rr2_rr1_R	TGTGGTTGATGCACATTATGGC^e^	7131–7152	
4	iap-3	OrNV_iap_3_F	GACGACGACTGTTGCGACTAC	17,129–17,149	722
		OrNV_iap_3_R	CGGTATTACGTACGTGGCGA	17,831–17,850	
5	hypothetical protein 22340	OrNV_hyp_prot_22340_F	CGGCAACTTTGTTGATCGCG^d^	19,738–19,757	781
		OrNV_hyp_prot_22340_R	TGTCCCCTACGTCTCCTACAC^e^	20,498–20,518	
6	19kDa-helicase	OrNV_19kDa_helicase_F	ATCGTCGCCGGTCAAATGTC^d^	31,984–32,003	828
		OrNV_19kDa_helicase_R	GTAAATGTATTGGCCGGGCAG^e^	32,791–32,811	
7^f^	Helicase (fragment 1)	OrNV_Helicase_1_F	CGCCAAGCTAAACGACGATG	34,138–34,157	1115
		OrNV_Helicase_1_R_2	CCGTGTTACCGTCACCCTG	35,234–35,252	
	Helicase (fragment 2)	OrNV_helicase_2_F	CAAATCAATCCCCATCCGCG	34,982–35,001	1085
		OrNV_helicase_2_R	GAACCAGGCTCTTCAATCACC	36,046–36,066	
8	hypothetical protein—61kAc	OrNV_hyp_prot_61kAc_F	GGGACGACGAACTACTCTG	37,062–37,080	1009
		OrNV_hyp_prot_61kAc_R	CTAAATGACGTTCAATCTAATTCTG	38,046–38,070	
9	gp97-23	OrNV_gp97_gp23_F	CGCCAACGAATAGACCGAC	40,839–40,857	1116
		OrNV_gp97_gp23_R	CAGAACGATACGCAACAGGC	41,935–41,954	
10	gp83	OrNV_gp83_F	ACGTTTAGCATCGCGGTGTA	49,991–50,010	1322
		OrNV_gp83_R	TTCTTCTTCGGCTCTGGGAG	51,293–51,312	
11^f^	gp28 (fragment 1)	OrNV_gp28_F2	ACTCTTCGATCGTGCTTTGTCG^d^	76,328–76,349	1270
		OrNV_gp28_R	GCCGACTACGATTTAACCGG^e^	77,578–77,597	
	gp28 (fragment 2)	OrNV_gp28_F3	AACGGTCGTCGGATAAATGCC	77,403–77,423	1049
		OrNV_gp28_R2	GAATTCGCCATCGTACGAGC	78,433–78,452	
12	ligase-gp39	OrNV_ligase-gp39_F	CTAGAGCCAGTATCACATATCGG	109,073–109,095	1202
		OrNV_ligase-gp39_R	AGGAGACAACGCAGCTTGAA	110,256–110,275	
13	hypothetical protein (114,000)	OrNV_hyp_prot_114000_F	CCGGCTTAGTCGTCGTAGTG	110,775–110,794	1215
		OrNV_hyp_prot_114000_R	TGCCAATACTTGATGGGAAACG	111,969–111,990	

^a^Location is given based on the nucleotide position in the OrNV X2B reference genome (MW298153)

^b^Personal communication Madoka Nakai (Tokyo University of Agriculture and Technology)

^c^Five overlapping primer sets were used to amplify the DNA polymerase B gene

^d^For ONT sequencing (PCR step 1), the primer contained barcode adapter 5ʹ-TTTCTGTTGGTGCTGATATTGC-3ʹ at the 5ʹ end

^e^For ONT sequencing (PCR step 1), the primer contained barcode adapter 5ʹ-ACTTGCCTGTCGCTCTATCTTC-3ʹ at the 3ʹ end

^f^Two overlapping primer sets were used to amplify the helicase and gp28 genes

### Amplicon generation for Sanger sequencing

To test all 19 primer sets, PCR amplification was performed for 17 OrNV isolates from the AgResearch Microbial Culture Collection (Table 1) in 20 μL reactions using 1 μL of purified viral DNA, 1 U MyTaq™ HS DNA Polymerase (Bioline, Meridian Biosciences, UK), or MyFi DNA Polymerase (Bioline, Meridian Biosciences, UK), 3 mM MgCl_2_ and final concentrations of 0.4–0.8 μM for the primer pairs used (Table 2). Initial denaturation was performed at 95 °C for 5 min, followed by 35 cycles of 30 s at 94 °C, 30 s annealing at 50 °C, 30 s at 72 °C for shorter amplicons or 1 min for longer amplicons, with a final 2 min extension at 72 °C. Amplifications were carried out in a Mastercycler thermocycler (Eppendorf, Germany). PCR products were resolved by electrophoresis in 1.2% agarose gels containing RedSafe™ (iNtRON, South Korea) DNA stain. Amplicons were purified and Sanger sequenced by Macrogen (Macrogen, South Korea). Sequence data received were trimmed, and consensus sequences were generated using Geneious Prime 2023.0.4 software (Kearse et al. 2012).

### OrNV classification based on either a single gene (DNA polymerase B) or multiple genes of OrNV

To evaluate the suitability of the DNA polymerase B gene (DNA pol B) for distinguishing between different OrNV isolates, we utilized Geneious Prime software (Kearse et al. 2012) with default settings. DNA pol B sequences, each trimmed to 2630 base pairs, were aligned for this analysis. This includes the 17 OrNV in Table 1 and additional OrNV DNA pol B sequences available in GenBank (*n* = 14) (Wang et al. 2007). From the alignment of the 31 OrNV DNA pol B sequences, a Neighbor-Joining tree was built with Geneious Prime Tree Builder using a Jukes-Cantor genetic distance model and 1000 bootstrap replicates. To demonstrate the variability of isolates, the distance method was used as it was sufficient to separate many, if not all, individual isolates based on the limited sequence variation present. Tree editing was performed in Mega 11.0.13 software (Tamura et al. 2021) and Corel PaintShop Pro 21.0.0.119.

For comparison, an additional analysis of 13 gene regions targeted for OrNV isolate identification was carried out. OrNV isolate NSB was not included in this analysis because the initial DNA pol B sequences clearly differentiated this isolate from the others. The remaining 16 OrNV isolates from the AgResearch Microbial Culture Collection (Table 1), plus four published reference sequences (Solomon Islands—MN623374, Malaysia—NC_011588, Palau—MW298154 and Indonesia—MZ727584) were used for this analysis. Each individual sequence fragment was trimmed to the same length, and trimmed fragments from each isolate or published reference sequence were concatenated, giving a total concatenated fragment length of 14,704 bp. Concatenated sequences were aligned, and the Neighbor-Joining tree was built as described above.

Finally, a similar analysis was performed for four selected amplicons (rr2_rr1, 19kDa, 22,340—hypothetical protein and gp28_fragment 1) that were concatenated to a total length of 3915 bp.

### Amplicon generation, library preparation and nanopore sequencing

For nanopore sequencing of selected OrNV genes, amplicons were generated in two consecutive reactions. First, primers with tails at the 5ʹ ends complementary to barcode primers were used for PCR amplification (Table 2). PCRs were performed in 25 μL reactions using 1 μL of extracted DNA template from either pure OrNV isolates (Table 1) or CRB tissue samples (Table 3), 2.5 μL of 10 × PCR buffer (with 25 mM Mg^2+^), 2 μL of dNTP mixture (2.5 mM each), 0.125 μL of TaKaRa Taq HS Polymerase (5 U/μL; TaKaRa Bio, USA), and 1 μL of each primer (0.4 μM final concentration). Initial denaturation was performed at 95 °C for 5 min, followed by 35 cycles of 20 s at 95 °C, 30 s annealing at 55 °C, 30 s or 1 min extension at 72 °C, with a final 5 min extension at 72 °C. PCR products were diluted to a total volume of 100 μL with water, 180 μL of AMPure XP beads (Beckman Coulter, United States) was added and amplicons were purified according to the manufacturer’s protocol. The concentration of eluted DNA was measured on a Nanodrop spectrophotometer (NanoPhotometer NP80; Implen, Germany).

**Table 3:** List of environmental samples of CRB positive for OrNV tested in multilocus nanopore sequencing. The presence of specific SNPs was identified based on the reference genome of OrNV X2B (MW298153) and Table 4

Country	Site	Collection No	rr2_rr1^a^					22340^a^				19kDa^a^				gp28_1^a^							Isolate identification based on ONT sequencing
			5985	6091	6543	6680	7089	19,843	19,906	19,920	20,050	31,998	32,100	32,612	32,654	76,569	76,714	76,870	76,891	76,985	77,020	77,424	
			G	A	C	G	T	A	C	T	G	T	T	C	C	C	A	C	C	A	A	T	
Papua New Guinea	Madang	21–0029		G	T		C		T	C	A	C			T		G			G	C		PNG-related
Papua New Guinea	Port Moresby	15–009		G	T		C		T	C	A	C			T		G			G	C		PNG-related
Papua New Guinea	Morobe	21–0038		G	T		C		T	C	A	C			T		G			G	C		PNG-related
Papua New Guinea	New Ireland	16–058		G	T		C		T	C	A	C			T		G	A		G	C		PNG-related
Papua New Guinea	East New Britain	15–059		G	T		C		T	C	A	C			T		G			G	C		PNG-related
Solomon Islands	Guadalcanal	21–0429	A	G				C				C		T		T	G			G	C	C	S2A
Solomon Islands	Guadalcanal	20–1154	A	G				C				C		T		T	G			G	C	C	S2A
Solomon Islands	Guadalcanal	20–0532	A	G				C				C		T		T	G			G	C	C	S2A
Solomon Islands	Guadalcanal	20–0514	A	G				C				C		T		T	G			G	C	C	S2A
Solomon Islands	Guadalcanal	20–0538	A	G				C				C		T		T	G			G	C	C	S2A
Solomon Islands	Guadalcanal	21–0397		G		T						C					G			G	C		V23B
Palau	Aimeliik	14–021		G	T		C		T	C	A	C			T		G		T	G	C		Palau1 (MW298154)^b^
Palau	Aimeliik,	14–023		G	T		C		T	C	A	C			T		G		T	G	C		Palau1 (MW298154)^b^
Fiji	Taveuni	14–031		G	T		C		T	C	A	C			T		G			G	C		PNG-related
Indonesia	Riau	16–071		G			C					C	C				G			G	C		Indonesia (LiboV) (MT150137)^c^

^a^SNPs were assigned according to alignment with the X2B reference genome sequence (MW298153)

^b^Tanaka, S. et al. (2021) Confirmation of *Oryctes rhinoceros* nudivirus infections in G-haplotype coconut rhinoceros beetles (*Oryctes rhinoceros*) from Palauan PCR-positive populations. Scientific Reports, 11, pp. 1–12

^c^Kurnia et al. (2020) *Oryctes rhinoceros* nudivirus strain LiboV, complete genome. Microbiology Resource Announcements, 10, pp. 10–11

In the second step, purified ‘step 1’ PCR amplicons from each sample were re-amplified together using barcoded primers in order to add a distinctive barcode corresponding to a specific OrNV sample. PCR amplification was performed in a 25 μL reaction using 1 μL of template DNA (0.3 ng/μL), 1.5 μL barcode primer (EXP-PBC096, ONT, UK), 12.5 μL LongAmp Hot Start Taq 2× Master Mix. Initial denaturation was performed at 94 °C for 3 min followed by 30 cycles of 30 s at 94 °C, 30 s annealing at 62 °C, 1 min 20 s extension at 65 °C with a final 10 min extension at 65 °C. PCR products were purified using AMPure XP beads as described above and eluted with 120 μL of water.

Nanopore library preparation and sequencing was performed according to the manufacturer’s protocol for the rapid sequencing kit (SQK-RAD004; ONT, UK). The sequencing run was performed on a MinION MK1B device using a FLO-MIN106 (R9.4.1) flowcell. A 40 ng (approximately 50 fmol) aliquot of the library was loaded on the flowcell (the remainder of the library was stored) and the run was stopped after 4 h. The minimum targeted sequencing depth was 1000× per amplicon per sample. Base-calling was performed using Guppy v5.0.14, and data were subject to quality control using pycoQC version 2.5.2 (Leger and Leonardi 2019). Basecalled data was analyzed in the EPI2ME platform (Metrichor Ltd., UK), and output data (Binary Alignment Map (BAM)) was analyzed in Geneious Prime 2023.0.4. A minimum read length of 200 bases and a minimum read q-score of 9 was used during basecalling. The consensus sequences for each amplicon and each sample were extracted and aligned with Sanger sequences previously obtained to ascertain the accuracy of the nanopore sequencing.

## Results

### Identification based on OrNV Polymerase B gene sequence

**Fig. 2:**
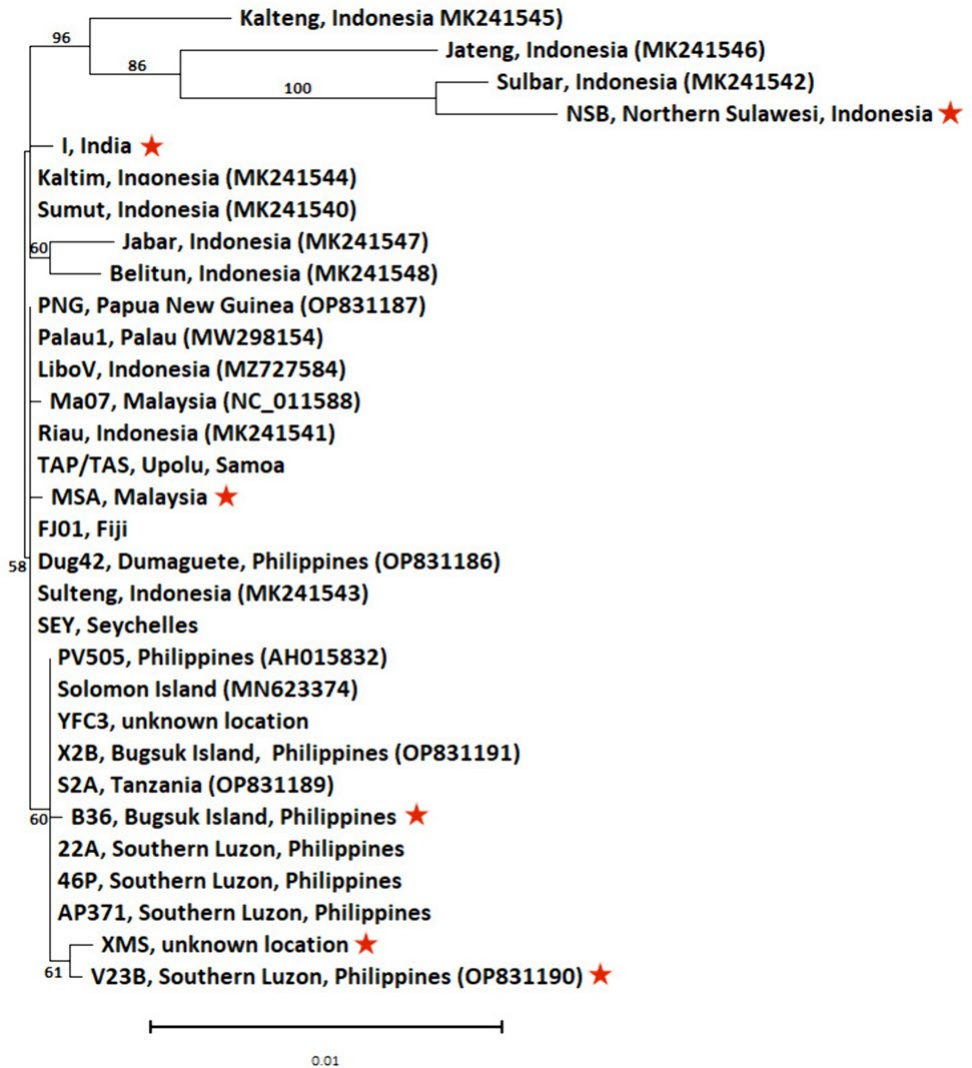
Visual representation of the resolution provided using only the DNA pol B gene to differentiate between the various OrNV isolates. Shown is a Neighbor-Joining tree based on alignment of the DNA polymerase B gene fragment (2630 bp) from different isolates of OrNV. GenBank accession numbers have been included in brackets after the isolate name. *Scale bar* represents the number of base substitutions per site. Bootstrap scores (proportion of 1000 replicates) over 50 are indicated. Individual OrNV isolates from the AgResearch Microbial Culture Collection that can be confidently distinguished based on the DNA pol B sequence have been marked with a *red star*

The DNA pol B gene has commonly been used as a marker to identify DNA viruses, and was investigated here as a possible marker for identifying between individual OrNV isolates (Wang et al. 2007). Using the partial DNA pol B gene sequence (2630 bp), Fig. 2 shows that the use of sequence information from the DNA pol B gene, both from isolates from our collection and from sequences in the public domain, provides a poor level of resolution to distinguish between individual OrNV isolates. Only four OrNV isolates (all from Indonesia: MK241545, MK241546, MK241542, and NSB) were able to be reliably resolved as a clade (branch support with >85% bootstrap support) based on the DNA pol B sequence. Of the 31 sequences assessed, only 12 individual OrNV isolates (including six from the AgResearch collection) could be distinguished from all other isolates based on the DNA pol B gene sequence fragment. The AgResearch isolates included NSB (Northern Sulawesi, Indonesia), I (India), MSA (Malaysia), B36 (Bugsuk Island, Philippines), V23B (Southern Luzon, Philippines), and XMS (unknown location). This demonstrated that the gene for DNA polymerase B does not have sufficient genetic variability to distinguish between individual OrNV isolates.

### Multilocus sequence analysis of OrNV

**Fig. 3:**
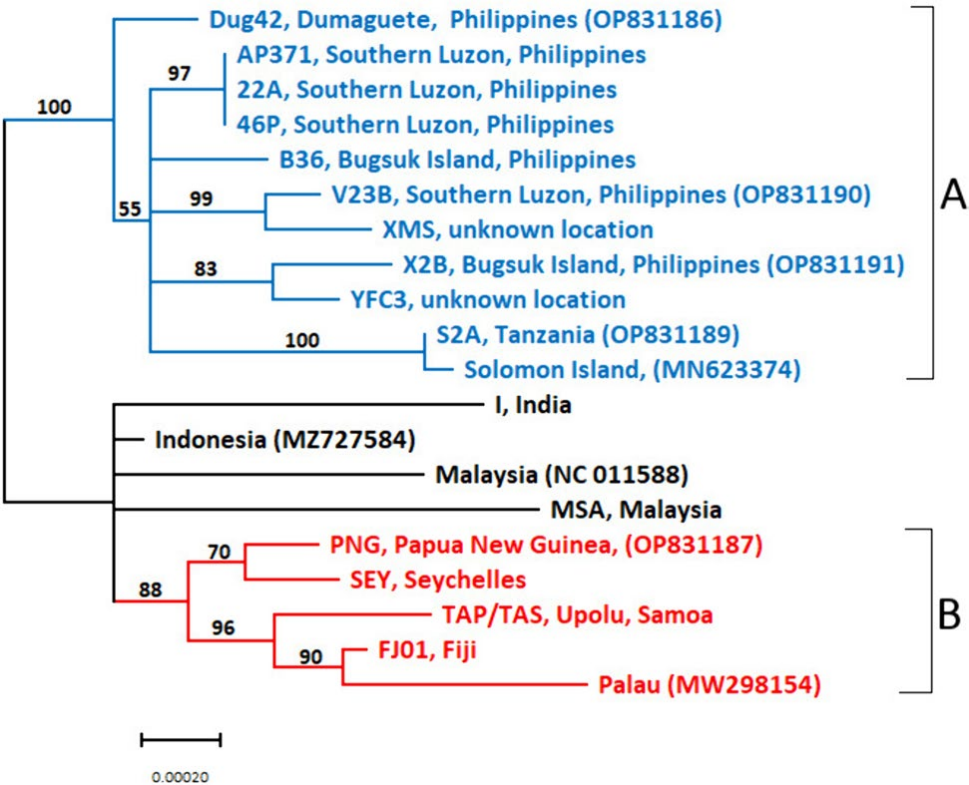
Visual representation of the resolution provided using 13 regions of the OrNV genome to differentiate between the various OrNV isolates. Shown is a Neighbor-Joining tree based on alignment of concatenated fragments (totaling 14,704 bp) from 13 OrNV genome regions analyzed for 16 OrNV isolates from the AgResearch Microbial Culture Collection and four published reference genomes. GenBank accession numbers have been included in brackets after the isolate name. *Scale bar* represents the number of base substitutions per site. Bootstrap scores (proportion of 1000 replicates) over 50 are indicated. Two major clades with >85 bootstrap scores (**A** and **B**) are indicated. *Blue branches* highlight the group A clade, *red branches* highlight the group B clade, and *black branches* indicate unresolved taxa

The low level of sequence variation found within the DNA pol B gene indicated the necessity of searching for genetic variation within other OrNV genes/regions for diagnostic purposes. We identified 100 polymorphic sites within 14,704 bp of concatenated sequence. The majority of those were SNPs that were identified within sequences of individual isolates. Analysis of the 13 concatenated genes/regions revealed two broad groupings consisting of isolates from different geographical regions (Fig. 3).

Group A forms a clade (100% bootstrap support) consisting of OrNV isolates from Philippines (22A, 46P, AP371, B36, Dug42, V23B, X2B), Solomon Islands (MN623374), Tanzania (S2A) and two isolates from unknown locations (XMS and YFC3). Group B forms a clade (88% bootstrap support) contains OrNV isolates from Palau (Palau1 [MW298154]), Samoa (TAP/TAS), Fiji (FJ01), Papua New Guinea (PNG) and Seychelles (SEY). All of these isolates come from the CRB invaded regions where subsequent introduction of OrNV as a classical biocontrol agent occurred. There were also four unresolved OrNV isolates collected from Malaysia (Ma07 [NC_011588] and MSA), Indonesia (LiboV [MZ727584]) and India (I); all of these locations are from the native range of CRB.

The concatenation of 13 different genome regions permitted most of the 20 OrNV isolates to be differentiated from each other (Fig. 3) based on unique SNP combinations (including 16 from the AgResearch collection; Table S1). As expected, some sequence regions were more variable than others. Using isolates from the AgResearch collection as an example, based on the 19kDa amplicon sequence, unique SNPs can be identified for isolates X2B, MSA, I, TAP/TAS and FJ01. Similarly, unique SNPs in the rr2_rr1 gene sequence can identify S2A, V23B, I and MSA. However, SNPs in the sequence of gene 61 kAc gave lower resolution, and 16 AgResearch isolates could only be divided into four groups, with the India isolate (I) having a unique SNP at position 37,168 (Table S1). Some isolates, like isolates 22A, 46P and AP371 (with each isolate corresponding to infected beetles from a different geographic origin in Southern Luzon, Philippines) could not be individually separated based on the OrNV genomic regions selected for isolate identification (100% sequence identity across 14,704 bp). The clustering of the Solomon Islands isolate (MN623374) with isolate S2A was supported by 100% bootstrap value. Our analysis revealed 99.9% identity across 14,704 bp suggesting that they are closely related. Overall, using a combination of SNPs identified from across all 13 genes/gene regions made differentiation between 17 of the 20 individual isolates possible.

### Viral isolate identification using nanopore sequencing

**Fig. 4:**
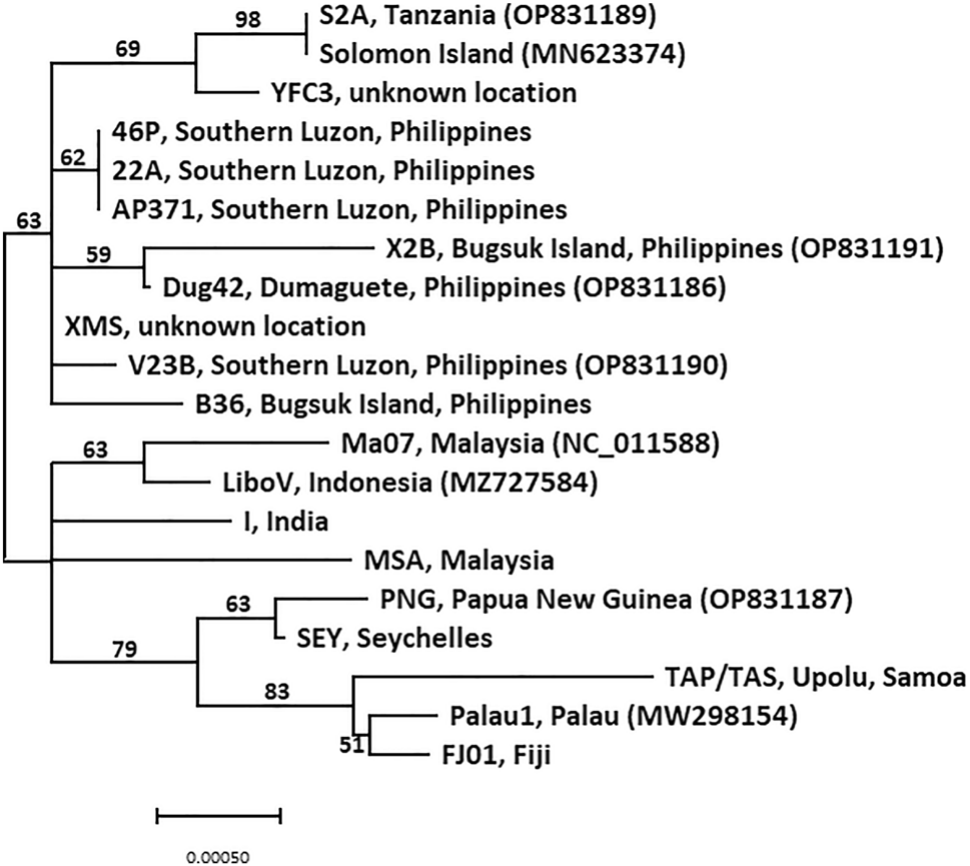
Visual representation of the resolution provided using four regions of the OrNV genome to differentiate between the various OrNV isolates. Shown is a Neighbor-Joining tree based on alignment of concatenated fragments (totaling 3915 bp) from four OrNV genome regions analyzed (rr2_rr1, 19kDa protein, 22,340 (position of hypothetical protein) and gp28 (fragment 1) for 16 OrNV isolates from the AgResearch Microbial Culture Collection and four published reference genomes (MN623374, NC_011588, MZ727584, MW298154). GenBank accession numbers have been included in brackets after the isolate name. *Scale bar* represents the number of base substitutions per site. Bootstrap scores (proportion of 1000 replicates) over 50 are indicated

To further streamline the process of identifying between different OrNV isolates (i.e., reduce the time and cost involved), we focused on fewer amplicons. Four genes were identified for multilocus sequence analysis (rr2_rr1, 22,340 (hypothetical protein location), 19kDa protein and gp28_fragment 1; Fig. 1). Despite the low branch support scores (Fig. 4), assessment based on key SNPs (Table 4) within these four regions was found to sufficiently distinguish between the OrNV isolates examined in this study (15 out of 20). Based on Sanger sequencing, the only isolates that can not be distinguished in the four amplicon assay were three isolates from Southern Luzon, Philippines (isolates 22A, 46P and AP371; 100% identity) and the two isolates S2A and Solomon Islands (100% identity).

**Table 4:**
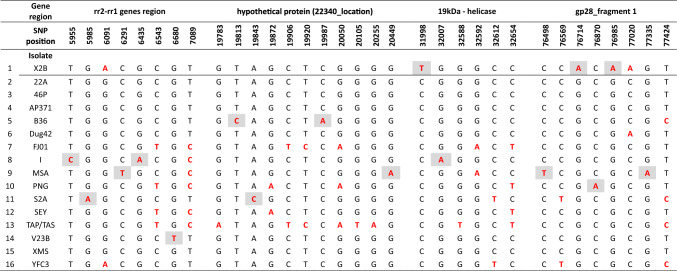
Unique SNP locations within sequences of the genes rr2_rr1, hypothetical protein (22340_location), 19kDa_helicase and gp28—fragment 1

SNP positions are calculated according to the X2B reference genome (MW298153). Red font highlights base variation from majority of 16 isolates examined and grey shading highlights those SNPs that are unique for a particular isolate

A multilocus OrNV isolate identification assay based on these four amplicons was tested for 16 AgResearch OrNV isolates using nanopore sequencing (ONT). The four selected fragments were amplified for each of the 16 isolates examined using primers containing barcode adapters (Table 2). All amplicons from one OrNV sample received the same barcode, and barcoded samples were pooled and sequenced, with coverage depth varying for individual amplicons and samples from 2000 to 15,000 reads.

The nucleotide position of each SNP was determined based on the genomic sequence of isolate X2B (MW298153) (Table 4). As an example, to identify isolate S2A, identification of SNPs from all four amplicons was needed: fragment rr2_rr1—unique G to A transition in position 5985; hypothetical protein 22340—unique A to C transversion in position 19,843; 19kDa gene—C to T transition in position 32,612; gene gp28—two unique transitions, C to T and T to C in positions 76,569 and 77,424, respectively.

### Validation of viral isolate identification from field collected samples of O. *rhinoceros*

The targeted nanopore sequencing method for OrNV strain identification was further validated using 15 field collected CRB specimens from five countries: Papua New Guinea, Solomon Islands, Fiji, Palau and Indonesia (Table 3). DNA from these CRB specimens had previously tested as OrNV positive (using PCR methods described by Richards et al. 1999). From each of these specimens, four amplicons (rr2_rr1; 19kDa; gp28_fragment 1 and 22,340-hypothetical protein region) were generated using ONT primers (Table 2). Depth of coverage varied for individual amplicons and samples, from 2000 to 8000 reads.

The consensus sequences were aligned to the reference genome (X2B; MW298153) to determine the presence of specific (unique) SNPs. Field collected viral samples were then identified based on the presence of those particular SNPs using Table 4. Based on these four OrNV sequences, we matched virus positive samples from Solomon Islands (*n* = 6) to isolate S2A (*n* = 5) and V23B (*n* = 1). Samples from Palau (*n* = 2) were matched to isolate Palau1, one sample from Fiji was matched as PNG-related and one from Indonesia was matched to the LiboV isolate. Samples from Papua New Guinea (*n* = 5) were matched as PNG-related (Table 3).

## Discussion

The aim of this study was to develop a method that allows accurate identification of OrNV isolates. This has become particularly important in light of the new populations of CRB detected in some invaded areas that have shown a degree of resistance towards some OrNV isolates commonly used as biological control agents (Jackson et al. 2005; Marshall et al. 2023). Such a method will be essential not only to search for new OrNV isolates (e.g. improved virulence and/or transmission), but to also monitor the spread of OrNV biocontrol agents after release to assist with CRB management efforts.

Improved affordability of DNA sequencing technologies is now allowing for whole OrNV genomes to be sequenced and for a more advanced characterization of viral isolates (Tanaka et al. 2021; Wang et al. 2008; Kurnia et al. 2021). We used published genomes of OrNV to design an assay initially focused on DNA pol B, a gene commonly used to reconstruct the evolutionary relationships of dsDNA viruses (Wang et al. 2007). We demonstrated that the DNA pol B gene is not variable enough to distinguish between all the OrNV isolates we tested. Analysis of the 2630 bp fragment of the DNA pol B gene for 17 OrNV isolates from our collection plus published sequences from Indonesia (Rahayuwati et al. 2020) and GenBank revealed overall poor separation between the majority of sequences. As the DNA pol B gene sequence analysis did not reveal sufficient variation, we expanded our assay to multilocus amplicon sequencing. Most importantly, the concatenation of the 13 selected gene fragments allowed differentiation between all major isolates based on key SNP variation. The exceptions being three (22A, 46P, and AP371) collected from within the Southern Luzon region of the Philippines that were found to be identical to each other over the 14,704 bp concatenated sequence.

Obtaining Sanger sequence for 19 amplicons (across 13 genes) for this analysis is time-consuming and costly. We have demonstrated that by careful selection of SNPs within four variable genes within the OrNV genome, we could confidently differentiate 15 out of 20 OrNV isolates tested, including those from the AgResearch Microbial Culture Collection, plus isolates such as Palau1 (Palau), LiboV (Indonesia) and Ma07 (Malaysia). Additionally, these four amplicons allow capture of de novo OrNV mutations within a CRB population and discovery of novel isolates in the field. The advantage of parallel sequencing of multiple amplicons of (barcoded) samples in one single experiment makes this method a lot more cost-effective and less time-consuming. This is particularly useful for fast throughput of field-collected CRB specimens. In the rare case where further discrimination between isolates is necessary, additional amplicon sequences from the 13 genes described above can be added to the analysis. The analysis of these 13 OrNV genes has given some insight into the potential origins of OrNV isolates and their use as biocontrol agents. We have found that isolates from Malaysia, Indonesia and India were unable to be resolved as a distinct clade, indicating a high level of diversity within OrNV as is anticipated within the native range for CRB (center of origin has been reported as SE Asia; Bedford 1980). Also of note were isolates from Fiji, Papua New Guinea, Palau, Samoa, and Seychelles displaying a close relationship. This reflects the pattern of reported OrNV releases, initiated with the introduction of OrNV as a biocontrol agent into the invasive range of CRB, starting with a Malaysian isolate (Huger 1966; precise provenance not reported) into Samoa in 1967 (Marshall 1970). Then virus collected from infected Samoan CRB was introduced to Fiji (1970–1974) (Bedford 1976), PNG (between 1978 and 1980) (Gorick 1980), Palau (1970, 1983) (Schreiner 1989) and Seychelles (1972) (Crawford et al. 1986). It appears that genomic changes have occurred in these isolates since the first virus introduction in Samoa. Additionally, we found that the sequences of isolates S2A (reported as originating in Samoa and passed through a different *Oryctes* species, *Oryctes monoceros*; Zelazny 1979), and the Solomon Islands isolate have 99.9% identity across the 13 gene regions studied. We believe that CRB samples collected from Guadalcanal (Solomon Islands) are highly likely to be infected with isolate S2A, as this was used in bioassays in Honiara from 2016 (Marshall et al., unpublished data).

Taking advantage of the versatility of nanopore sequencing, we designed an assay based on four OrNV amplicons. After the proof of concept using purified OrNV isolate DNA, we investigated field samples of CRB. Total DNA isolated from CRB midgut tissue was used for PCR and sequencing. We identified OrNV isolates by matching unique SNPs characteristic for specific OrNV isolates, both from our collection and published sequences.

Here, we designed a multiplex nanopore sequencing method using four selected OrNV amplicons for rapid and accurate OrNV isolate matching. It can be employed to identify the diversity of OrNV between geographic locations and assist with monitoring the establishment and distribution of OrNV isolates released into the field for biocontrol purposes. Additionally, it has potential to assist with the selection of the most effective isolates to be used against CRB populations occurring in different environmental zones. While currently there is a lack of empirical data allowing comparison of virulence and other OrNV characteristics, future research could be used in conjunction with this information to assess likelihood of successful biocontrol.

## Supplementary Information

Below is the link to the electronic supplementary material.

Supplementary file1 (XLSX 29 KB)

## Data Availability

The datasets generated and analysed during this study are available from GenBank with accession numbers PP871001–PP871208.
